# Association of Dietary Antioxidant Potential with Sarcopenia in Hypertension

**DOI:** 10.31083/RCM27138

**Published:** 2025-04-24

**Authors:** Yufan Wang, Li Liu, Shandi Yang, Bingquan Xiong, Xumin Xin

**Affiliations:** ^1^Department of Cardiovascular Medicine Intensive Care Unit, The Second Affiliated Hospital of Hainan Medical University, 570311 Haikou, Hainan, China; ^2^Department of Cardiology, The Second Affiliated Hospital of Chongqing Medical University, 400010 Chongqing, China

**Keywords:** diet, antioxidant, hypertension, sarcopenia, risk

## Abstract

**Background:**

The high prevalence of sarcopenia among hypertensive adults is a global health issue. Growing literature demonstrates that a high antioxidant diet can protect against sarcopenia. However, little attention has been paid to the association between the dietary composite antioxidant intake and sarcopenia in hypertension. To investigate the potential efficacy of the composite dietary antioxidant index (CDAI) on sarcopenia among hypertensive adults.

**Methods:**

This study included 6995 hypertensive adults from the National Health and Nutrition Examination Survey (NHANES) 2001–2006 and 2013–2018, with 3212 (45.92%) females and 3783 (54.08%) males. Appendicular lean mass (ALM) and sarcopenia were assessed by dual-energy X-ray absorptiometry (DEXA). All hypertensive adults participating in NHANES were eligible to participate in dietary interviews, and the average intake of six antioxidants over two days was used to calculate the CDAI. Logistic regression was conducted to determine odds ratios (ORs) and 95% confidence intervals (CIs). Subgroup analyses and restricted cubic spline (RCS) regressions were additionally utilized.

**Results:**

The mean age was 48.47 ± 0.27 years old, and 1059 (15.14%) were considered to have sarcopenia. The highest quartile had a 61% decreased risk of sarcopenia (OR = 0.39, 95% CI: 0.25, 0.60) compared with the lowest quartile of CDAI. RCS revealed a linear association between CDAI with sarcopenia and ALM. Subgroup analyses demonstrated a more pronounced inverse correlation between CDAI and sarcopenia in females.

**Conclusions:**

In summary, our results indicated a reverse correlation between CDAI and sarcopenia in hypertension. These findings highlighted the beneficial role of an antioxidant-rich diet in prevention and provided a valid method for managing sarcopenia in hypertensive adults.

## 1. Introduction 

Sarcopenia, a chronic condition marked by diminished muscle quality, compromises the quality of life and escalates medical burdens [1]. It is linked to a multitude of diseases. Prominent symptoms include weakness, fatigue, reduced energy, balance impairments, and difficulties with ambulation and standing [2]. A recent study has elucidated the high prevalence of sarcopenia among older adults, notably those with hypertension [3]. Ata and colleagues reported that the incidence of sarcopenia in hypertensive individuals is sixfold higher compared to normotensive counterparts [4]. A large-scale analysis of 15,779 American adults further corroborated that hypertension is intricately linked to an elevated prevalence of sarcopenia [5]. The disparity may be ascribed to the oxidative stress (OS) and chronic inflammation commonly observed in hypertensive patients, both of which are pivotal mechanisms driving the onset of sarcopenia. Additionally, several studies have confirmed that the accumulation of OS and reactive oxygen species (ROS) may contribute to age-related muscle loss [6, 7], while chronic inflammation can disrupt skeletal muscle protein synthesis, thereby increasing the incidence of sarcopenia [8]. Insulin resistance (IR) further influences muscle protein metabolism by interfering with multiple signaling pathways, exacerbating the decline of muscle mass [3]. Thus, effective control of OS, inflammation, and IR in subjects with hypertension may be a potential protective measure to reduce the occurrence of sarcopenia.

Adjusting dietary structure is a valid intervention method that can improve health status by reducing systemic OS levels [9]. Prior research has demonstrated that a higher intake of carotenoids and various vitamins is beneficial in mitigating age-related muscle loss [10]. Combined supplementation of vitamins D and E has been shown to significantly enhance muscle strength in older adults with sarcopenia [11]. A recent study found that adherence to the intake of vitamins A, C, E, and K was linked to a reduced risk of sarcopenia [12]. Another study also showed that increased consumption of dietary microelements had various positive effects on physical performance and decreased the risk of sarcopenia [13]. Conversely, inadequate intake of micronutrients led to diminished antioxidant capacity and declining muscle function [14]. Nevertheless, most previous literature has primarily focused on one or a few nutrients, with little attention given to the impact of overall dietary antioxidant capacity on sarcopenia. The Composite Dietary Antioxidant Index (CDAI) is a reliable assessment tool designed to reflect the total antioxidant capacity (TAC) of individuals, emphasizing the cumulative and synergistic effects of diverse dietary antioxidants [15]. However, few studies have discussed the antioxidant diet impacts on sarcopenia risk in hypertensive individuals. Thus, this study aimed to evaluate the association between CDAI and sarcopenia risk in hypertensive individuals, exploring dose-response relationships and gender differences. To address this knowledge gap, the National Health and Nutrition Examination Survey (NHANES) database was applied in the current research.

## 2. Materials and Methods

### 2.1 Study Design

The NHANES is a cross-sectional program established by the National Center for Health Statistics (NCHS) to investigate the prevalence and risk factors of common diseases in the United States. The NHANES protocol was approved by the NCHS Ethics Review Board, obviating the need for additional local ethical approval. On the whole, the data utilized in this analysis is publicly accessible through the NHANES online portal.

Our research leveraged publicly available data from six NHANES cycles (2001–2002, 2003–2004, 2005–2006, 2013–2014, 2015–2016, 2017–2018) to substantiate the true association between CDAI and sarcopenia in hypertension. Specifically, we surveyed 70,655 participants from NHANES 2001–2006 and 2013–2018, initially excluding participants without hypertension (n = 49,772) and those aged less than 20 years old (n = 891). And then, we excluded individuals with missing values for sarcopenia data (n = 10,583), CDAI data (n = 1106) or any other covariates data (n = 1187), and excessive daily energy intake (<500 or >5000 kcal per day) (n = 131). Finally, we analyzed 6995 eligible hypertensive adults in our study (**Supplementary Fig. 1**).

### 2.2 Diagnosis of Hypertension 

NHANES provided a standardized protocol and procedure for blood pressure measurement. Highly trained examiners conducted three or four blood pressure measurements in mobile examination center (MEC) or during home visits, with participants resting quietly for five minutes before determining the maximum inflation level. To minimize bias, we calculated the average blood pressure to obtain the final blood pressure recording for subsequent statistical analysis [16]. In the current study, hypertension was diagnosed based on self-reported physician diagnoses, a history of antihypertensive medication use, or systolic blood pressure (SBP) ≥130 mmHg and/or diastolic blood pressure (DBP) ≥80 mmHg [17].

### 2.3 Definition of Sarcopenia 

The appendicular lean mass (ALM) can be obtained from the NHANES database, which was measured by dual-energy X-ray absorptiometry. Then, the sarcopenia index was obtained by dividing the ALM (kg) by the body mass index (BMI, kg/m^2^), and sarcopenia was diagnosed based on sex-specific sarcopenia index (cut-off value: 0.512 for females and 0.789 for males) [18].

### 2.4 Assessment of CDAI

The CDAI consisted of six antioxidants such as vitamins A, C, E, zinc, selenium, and carotenoids. Each antioxidant was standardized by subtracting the total mean and dividing by the total standard deviation (SD). Subsequently, we summarized the standardized intake of individual nutrients according to the formula reported in previous research to derive the CDAI [19]. The detailed calculations can be found in **Supplementary Table 1**.

### 2.5 Covariates

According to related research, we considered the following covariates that are associated with sarcopenia: Age, gender, race, marital status, education level, poverty income ratio (PIR) [20], BMI [21], smoking status [22], alcohol consumption [23], cardiovascular disease (CVD), chronic kidney disease (CKD), diabetes [24], uric acid [25], blood urea nitrogen (BUN) [26], white blood cell (WBC) [27], hyperlipidemia [28], and daily energy intake [29] were considered as potential covariates associated with sarcopenia. More details of these covariates are presented in **Supplementary Table 2**.

### 2.6 Statistical Analysis

Following the NHANES analysis guidelines, we applied the appropriate sampling weights and accounted for the complex survey design to ensure unbiased estimates in all analyses. Differences in baseline characteristics among the four groups were assessed using one-way analysis of variance (ANOVA) for continuous variables and chi-square tests for categorical variables. Continuous variables were presented as mean ± standard error (SE), and category variables were presented as the frequency with percentage. Logistic regression models were built to estimate the odds ratios (ORs) and 95% confidence intervals (CIs) between CDAI and sarcopenia after adjusting for confounding factors. Likewise, we further explored the relationship between CDAI and ALM using linear regression. Model 1 was an unadjusted crude model. Model 2 was adjusted for age, gender, and race. Model 3 further refined Model 2 by including adjustments for marital status, PIR, education level, BMI, smoking, alcohol use, CVD, CKD, diabetes, uric acid, BUN, WBC, hyperlipidemia, and energy intake. In these models, we converted CDAI from a continuous variable into a categorical variable to verify the CDAI-sarcopenia association. Moreover, a linear trend was tested by entering CDAI as an ordinal variable. Notably, we also assessed the ORs with 95% CIs for each 1-SD increase across different models. To further illustrate the association between sarcopenia, ALM, and CDAI, we conducted restricted cubic spline (RCS) analysis. Notably, the risk of sarcopenia and daily intake of individual antioxidants were also assessed in both minimally adjusted, and fully adjusted models.

Subgroup analyses stratified by age, gender, BMI, diabetes, race, PIR, and alcohol status were performed to investigate the correlation between CDAI and sarcopenia in different populations. The likelihood ratio test was applied to check the interaction among these subgroups.

Additionally, several sensitivity analyses were conducted to assess the robustness of the associations between CDAI and sarcopenia [30]. A new hypertensive cut-off value of 140/90 mmHg was selected to verify the results in the above statistical models. Next, considering that each diagnostic criterion has its advantages and limitations, we used three hypertension diagnosis criteria to verify the correlation between CDAI and sarcopenia risk among hypertensive adults.

Subjects with missing data were excluded from our research. The significance level was set at α = 0.05, and all statistical tests were two-tailed. Statistical analyses were performed using R software (version 4.1.3, R Foundation for Statistical Computing, Vienna, Austria).

## 3. Results

### 3.1 Baseline Characteristics of the Participants

Table 1 shows the baseline characteristics of the study population stratified by the CDAI quartiles. The study included 6995 hypertensive adults from NHANES 2001–2006 and 2013–2018, with 3212 (45.92%) females and 3783 (54.08%) males. The mean age of enrolled participants was 48.47 ± 0.27 years old, and 1059 (15.14%) were considered to have sarcopenia. Compared with those in the lowest quartile (Q1), participants in the highest quartile (Q4) were more likely to be Non-Hispanic White, college-educated, not-impoverished, married, non-smokers, mild/heavy drinkers, to have a higher energy intake, had lower WBC levels, and had higher BUN levels. Moreover, they were less likely to have CKD, CVD, and sarcopenia. No significant differences were observed in age, gender, uric acid, diabetes, and hyperlipidemia between the four groups at baseline.

**Table 1.** **Characteristics of study participants by CDAI quartiles, weighted (n = 6995) ^𝐚^**.

Characteristic		Overall	Q1	Q2	Q3	Q4	*p* value
Sample		6995	1749	1748	1749	1749	
Age, years		48.47 ± 0.27	49.20 ± 0.52	49.07 ± 0.48	48.35 ± 0.45	47.42 ± 0.49	0.073
Gender, n (%)							0.081
	Female	3212 (45.92)	815 (48.77)	766 (43.80)	785 (42.23)	846 (46.00)	
	Male	3783 (54.08)	934 (51.23)	982 (56.20)	964 (57.77)	903 (54.00)	
Race, n (%)							0.035
	Mexican American	1117 (15.97)	289 (6.96)	305 (7.63)	267 (7.35)	256 (6.09)	
	Non-Hispanic Black	1591 (22.74)	470 (15.45)	380 (11.25)	357 (11.02)	384 (11.17)	
	Non-Hispanic White	3278 (46.86)	771 (65.79)	828 (70.81)	843 (69.80)	836 (70.52)	
	Other races	1009 (14.42)	219 (11.81)	235 (10.30)	282 (11.82)	273 (12.22)	
Education, n (%)							<0.001
	Less than 9th grade	708 (10.12)	277 (6.53)	179 (4.89)	137 (4.36)	115 (2.43)	
	9th–11th grade	951 (13.60)	301 (14.77)	255 (11.38)	205 (9.28)	190 (7.16)	
	High school	1708 (24.42)	443 (27.92)	442 (28.61)	430 (24.33)	393 (21.66)	
	Some college	2088 (29.85)	504 (32.91)	510 (30.80)	534 (33.41)	540 (31.92)	
	College or above	1540 (22.02)	224 (17.88)	362 (24.31)	443 (28.61)	511 (36.82)	
PIR, n (%)							<0.001
	<1.30	1892 (27.05)	606 (26.20)	479 (19.38)	419 (16.86)	388 (15.57)	
	1.30–3.49	2685 (38.38)	701 (39.82)	670 (33.94)	687 (38.19)	627 (31.31)	
	≥3.50	2418 (34.57)	442 (33.98)	599 (46.68)	643 (44.95)	734 (53.12)	
Marital status, n (%)							0.037
	Unmarried	981 (14.02)	241 (14.21)	218 (12.96)	263 (14.30)	259 (14.01)	
	Married	3981 (56.91)	935 (55.59)	1015 (60.48)	1002 (58.75)	1029 (63.22)	
	Others	2033 (29.06)	573 (30.20)	515 (26.56)	484 (26.94)	461 (22.78)	
Energy, kcal/d		2148.82 ± 19.43	1479.97 ± 18.68	1948.64 ± 21.86	2323.33 ± 25.53	2719.88 ± 31.24	<0.001
WBC, 1000 cell/µL		7.44 ± 0.05	7.59 ± 0.10	7.49 ± 0.09	7.50 ± 0.08	7.23 ± 0.07	0.004
Uric acid, µmol/L		338.86 ± 1.44	340.83 ± 2.99	343.66 ± 2.70	338.41 ± 2.92	333.20 ± 2.96	0.101
BUN, mmol/L		4.82 ± 0.03	4.60 ± 0.07	4.82 ± 0.07	4.85 ± 0.06	4.98 ± 0.06	<0.001
Smoke, n (%)		1550 (22.16)	510 (30.67)	390 (24.42)	363 (22.43)	287 (15.20)	<0.001
CVD, n (%)		815 (11.65)	254 (10.70)	220 (10.28)	183 (9.03)	158 (7.00)	0.020
CKD, n (%)		1316 (18.81)	410 (19.18)	344 (15.10)	308 (13.85)	254 (11.40)	<0.001
Diabetes, n (%)		966 (13.81)	253 (10.72)	247 (12.44)	234 (9.66)	232 (9.57)	0.132
Hyperlipidemia, n (%)		5452 (77.94)	1387 (77.32)	1379 (77.78)	1370 (80.47)	1316 (76.99)	0.332
Alcohol status, n (%)							0.012
	Never	906 (12.95)	267 (13.93)	209 (9.66)	215 (9.97)	215 (9.74)	
	Former	1269 (18.14)	381 (16.29)	338 (16.28)	272 (14.36)	278 (13.04)	
	Mild	2434 (34.80)	508 (31.54)	641 (39.46)	642 (36.75)	643 (37.47)	
	Heavy	2386 (34.11)	593 (38.24)	560 (34.61)	620 (38.91)	613 (39.75)	
Sarcopenia, n (%)		1059 (15.14)	343 (17.55)	306 (13.63)	228 (10.21)	182 (6.60)	<0.001

Continuous variables were shown as mean ± SE, categorical variables were shown as frequency (percentage). ^a^All estimates accounted for complex survey designs, and all percentages were weighted. Q1: CDAI ≤–2.83; Q2: –2.83 < CDAI ≤ –0.47; Q3: –0.47 < CDAI ≤ 1.87; Q4: CDAI >1.87. Abbreviations: Q, quartiles; SE, standard error; CDAI, composite dietary antioxidant index; PIR, poverty income ratio; WBC, white blood cell; BUN, blood urea nitrogen; CVD, cardiovascular disease; CKD, chronic kidney disease.

### 3.2 Association between CDAI and ALM/Sarcopenia

**Fig. 1.**
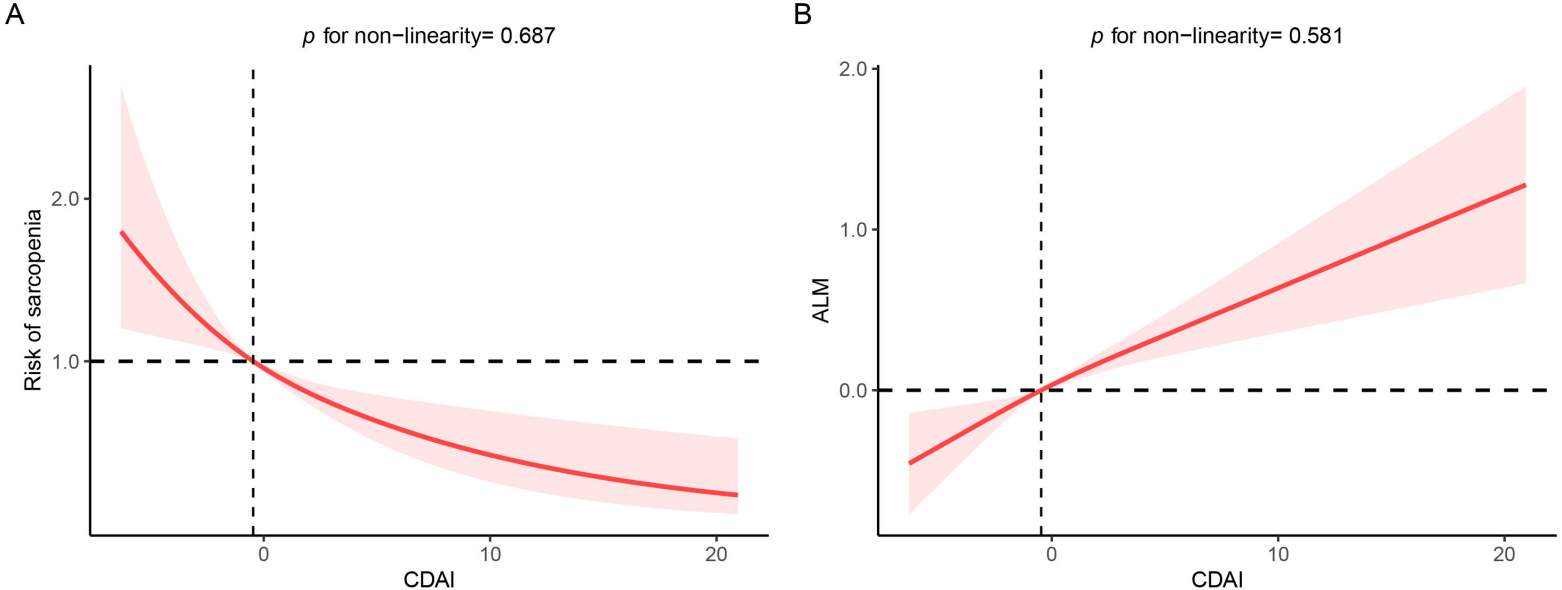
**Restricted cubic spline (RCS) analysis with multivariate-adjusted association between CDAI and ALM/sarcopenia**. (A) RCS analysis between CDAI and sarcopenia risk. (B) RCS analysis between CDAI and ALM risk. Abbreviations: ALM, appendicular lean mass; CDAI, composite dietary antioxidant index.

The associations between CDAI and ALM/sarcopenia are shown in Table 2. Overall, there was an inverse association between CDAI and sarcopenia whereas a positive association between CDAI and ALM in the three statistical models was shown. In the fully adjusted model, the adjusted ORs (95% CIs) were 0.75 (0.57–0.99) for Q2, 0.53 (0.36–0.77) for Q3, and 0.39 (0.25–0.60) for Q4 compared to Q1. Similar results were found when CDAI was treated as a continuous variable. Moreover, eacb 1 SD increase in CDAI was related to a 28% reduced risk of sarcopenia. We further employed multivariable linear regression to explore the relationship between CDAI and ALM. After controlling all confounders, a higher CDAI level was linked to increased ALM, and the corresponding β (95% CI) of Q4 was 0.54 (0.20, 0.87) when we selected Q1 as the reference. The results of the per 1 SD increase supported our findings in the same model. Importantly, *p* values for trend were less than 0.05 in the fully adjusted logistic or linear regression models. Additionally, RCS displayed a linear relationship of CDAI with sarcopenia and ALM (both *p* for non-linearity >0.050), suggesting a protective effect on sarcopenia at higher CDAI exposure ranges (Fig. 1).

**Table 2.** **The association between CDAI and sarcopenia risk among hypertensive adults**.

Variables			Model 1		Model 2		Model 3	
			β/OR (95% CI)	*p* value	β/OR (95% CI)	*p* value	β/OR (95% CI)	*p* value
ALM (kg)							
	Continuous		0.10 (0.04, 0.16)	<0.001	0.12 (0.09, 0.16)	<0.001	0.06 (0.03, 0.10)	<0.001
	Categories							
		Q1	Reference		Reference		Reference	
		Q2	0.95 (0.32, 1.59)	0.004	0.65 (0.26, 1.04)	0.001	0.22 (–0.07, 0.50)	0.137
		Q3	1.41 (0.68, 2.14)	<0.001	1.03 (0.57, 1.48)	<0.001	0.34 (0.03, 0.66)	0.032
		Q4	1.38 (0.77, 1.99)	<0.001	1.36 (0.97, 1.76)	<0.001	0.54 (0.20, 0.87)	0.002
	*p* for trend		<0.001		<0.001		0.004	
	Per 1-SD increase		0.38 (0.16, 0.59)	<0.001	0.46 (0.31, 0.60)	<0.001	0.24 (0.12, 0.36)	<0.001
Sarcopenia								
	Continuous		0.89 (0.86, 0.92)	<0.001	0.89 (0.85, 0.92)	<0.001	0.92 (0.86, 0.97)	0.003
	Categories							
		Q1	Reference		Reference		Reference	
		Q2	0.75 (0.60, 0.93)	0.010	0.71 (0.57, 0.88)	0.003	0.75 (0.57, 0.99)	0.043
		Q3	0.55 (0.41, 0.72)	<0.001	0.51 (0.39, 0.68)	<0.001	0.53 (0.36, 0.77)	0.001
		Q4	0.35 (0.26, 0.47)	<0.001	0.34 (0.25, 0.45)	<0.001	0.39 (0.25, 0.60)	<0.001
	*p* for trend		<0.001		<0.001		<0.001	
	Per 1-SD increase		0.65 (0.57, 0.75)	<0.001	0.64 (0.56, 0.74)	<0.001	0.72 (0.59, 0.89)	0.003

Model 1: Adjusted for age. Model 2: Adjusted for age, gender, race. Model 3: Adjusted for age, gender, race, marital status, education level, PIR, BMI, smoke, alcohol status, CVD, CKD, diabetes, uric acid, BUN, WBC, hyperlipidemia, and energy. Abbreviations: Q, quartiles; CDAI, composite dietary antioxidant index; PIR, poverty income ratio; WBC, white blood cell; BUN, blood urea nitrogen; CVD, cardiovascular disease; CKD, chronic kidney disease; BMI, body mass index; ALM, appendicular lean mass; OR, odds ratio; CI, confidence interval; SD, standard deviation.

### 3.3 Individual Antioxidant Component and ALM/Sarcopenia

A correlation between specific antioxidant constituents and sarcopenia/ALM were assessed by dividing the value of each individual antioxidant into quartiles, with Q1 selected as the reference category. The detailed results are shown in Table 3. After adjusting all confounding factors, for ALM, the β (95% CI) comparing Q4 with Q1 was 0.30 (0.01, 0.59) for vitamin A, 0.59 (0.22, 0.95) for vitamin E, 0.35 (0.10, 0.59) for vitamin C, and 0.33 (0.04, 0.62) for carotenoids, respectively. For sarcopenia, inverse associations of sarcopenia with vitamin A, vitamin E, vitamin C, and carotenoids were observed in the fully adjusted model. When comparing with Q1, Q4 of vitamin A, vitamin E, vitamin C, and carotenoids were all linked to a lower risk of sarcopenia among hypertensive adults, with corresponding ORs (95% CIs) of 0.67 (0.47, 0.97), 0.48 (0.31, 0.74), 0.71 (0.48, 0.91), and 0.72 (0.51, 0.92). All *p* values for the trend in the above regression results were significant. Lastly, the RCS of individual antioxidants and sarcopenia are displayed in **Supplementary Fig. 2**. We observed that vitamin A, E, and carotenoids were all linearly associated with sarcopenia risk, whereas a non-linear relationship was found between vitamin C and sarcopenia.

**Table 3.** **Multivariable-adjusted regressions according to daily intake of an individual antioxidant component**.

		Sarcopenia				ALM (kg)			
		Mini-adjusted model		Fully adjusted model		Mini-adjusted model		Fully adjusted model	
		OR (95% CI)	*p* value	OR (95% CI)	*p* value	β (95% CI)	*p* value	β (95% CI)	*p* value
Vitamin A									
	Q1	Reference		Reference		Reference		Reference	
	Q2	0.93 (0.70, 1.25)	0.637	1.03 (0.74, 1.43)	0.880	0.80 (0.13, 1.48)	0.019	0.14 (–0.17, 0.46)	0.373
	Q3	0.66 (0.49, 0.89)	0.007	0.78 (0.57, 1.06)	0.115	0.97 (0.31, 1.63)	0.004	0.29 (0.03, 0.54)	0.026
	Q4	0.49 (0.35, 0.67)	<0.001	0.67 (0.47, 0.97)	0.036	1.47 (0.74, 2.20)	<0.001	0.30 (0.01, 0.59)	0.044
	*p* for trend	<0.001		0.014		<0.001		0.033	
Vitamin E									
	Q1	Reference		Reference		Reference		Reference	
	Q2	0.67 (0.51, 0.89)	0.007	0.80 (0.57, 1.10)	0.168	1.02 (0.44, 1.60)	<0.001	0.26 (–0.04, 0.56)	0.091
	Q3	0.63 (0.47, 0.84)	0.002	0.76 (0.54, 1.08)	0.126	1.70 (1.12, 2.28)	<0.001	0.31 (0.04, 0.58)	0.024
	Q4	0.39 (0.28, 0.53)	<0.001	0.48 (0.31, 0.74)	0.001	3.21 (2.57, 3.86)	<0.001	0.59 (0.22, 0.95)	0.002
	*p* for trend	<0.001		0.003		<0.001		0.006	
Vitamin C									
	Q1	Reference		Reference		Reference		Reference	
	Q2	0.95 (0.76, 1.19)	0.643	1.15 (0.87, 1.52)	0.334	0.59 (–0.09, 1.28)	0.086	0.10 (–0.15, 0.36)	0.428
	Q3	0.62 (0.45, 0.84)	0.002	0.75 (0.54, 0.86)	0.024	0.99 (0.44, 1.54)	<0.001	0.35 (0.08, 0.62)	0.012
	Q4	0.57 (0.41, 0.78)	<0.001	0.71 (0.48, 0.91)	0.026	1.17 (0.61, 1.72)	<0.001	0.35 (0.10, 0.59)	0.006
	*p* for trend	<0.001		0.023		<0.001		0.002	
ZinC									
	Q1	Reference		Reference		Reference		Reference	
	Q2	1.05 (0.79, 1.41)	0.717	1.06 (0.75, 1.48)	0.745	1.35 (0.76, 1.94)	<0.001	–0.02 (–0.30, 0.26)	0.872
	Q3	0.76 (0.52, 1.12)	0.163	0.83 (0.53, 1.31)	0.424	2.82 (2.26, 3.38)	<0.001	0.01 (–0.33, 0.35)	0.940
	Q4	0.62 (0.45, 0.87)	0.006	0.66 (0.40, 1.08)	0.100	4.69 (4.07, 5.31)	<0.001	0.28 (–0.09, 0.65)	0.141
	*p* for trend	0.003		0.075		<0.001		0.187	
Selenium									
	Q1	Reference		Reference		Reference		Reference	
	Q2	0.72 (0.52, 1.00)	0.047	0.68 (0.47, 0.99)	0.045	1.42 (0.90, 1.94)	<0.001	–0.06 (–0.32, 0.21)	0.675
	Q3	0.73 (0.54, 1.01)	0.054	0.74 (0.49, 1.11)	0.140	3.31 (2.77, 3.84)	<0.001	–0.09 (–0.39, 0.22)	0.559
	Q4	0.59 (0.44, 0.79)	<0.001	0.66 (0.43, 1.03)	0.064	5.19 (4.55, 5.84)	<0.001	0.34 (–0.05, 0.73)	0.085
	*p* for trend	0.001		0.107		<0.001		0.141	
Carotenoids									
	Q1	Reference		Reference		Reference		Reference	
	Q2	0.99 (0.75, 1.29)	0.920	0.97 (0.70, 1.35)	0.863	0.17 (–0.38, 0.72)	0.545	0.07 (–0.22, 0.36)	0.647
	Q3	0.92 (0.69, 1.22)	0.571	0.92 (0.66, 1.27)	0.593	0.5 (–0.13, 1.13)	0.119	0.18 (–0.10, 0.46)	0.200
	Q4	0.61 (0.45, 0.82)	0.001	0.72 (0.51, 0.92)	0.033	1.02 (0.42, 1.61)	0.001	0.33 (0.04, 0.62)	0.025
	*p* for trend	0.002		0.039		0.001		0.017	

Mini-adjusted model: Adjusted for age. Fully-adjusted model: Adjusted for age, gender, race, marital status, education level, PIR, BMI, smoke, alcohol status, CVD, CKD, diabetes, uric acid, BUN, WBC, hyperlipidemia and energy. Abbreviations: Q, quartiles; PIR, poverty income ratio; WBC, white blood cell; BUN, blood urea nitrogen; CVD, cardiovascular disease; CKD, chronic kidney disease; BMI, body mass index; ALM, appendicular lean mass; OR, odds ratio; CI, confidence interval.

### 3.4 Subgroup and Sensitivity Analysis

**Fig. 2.**
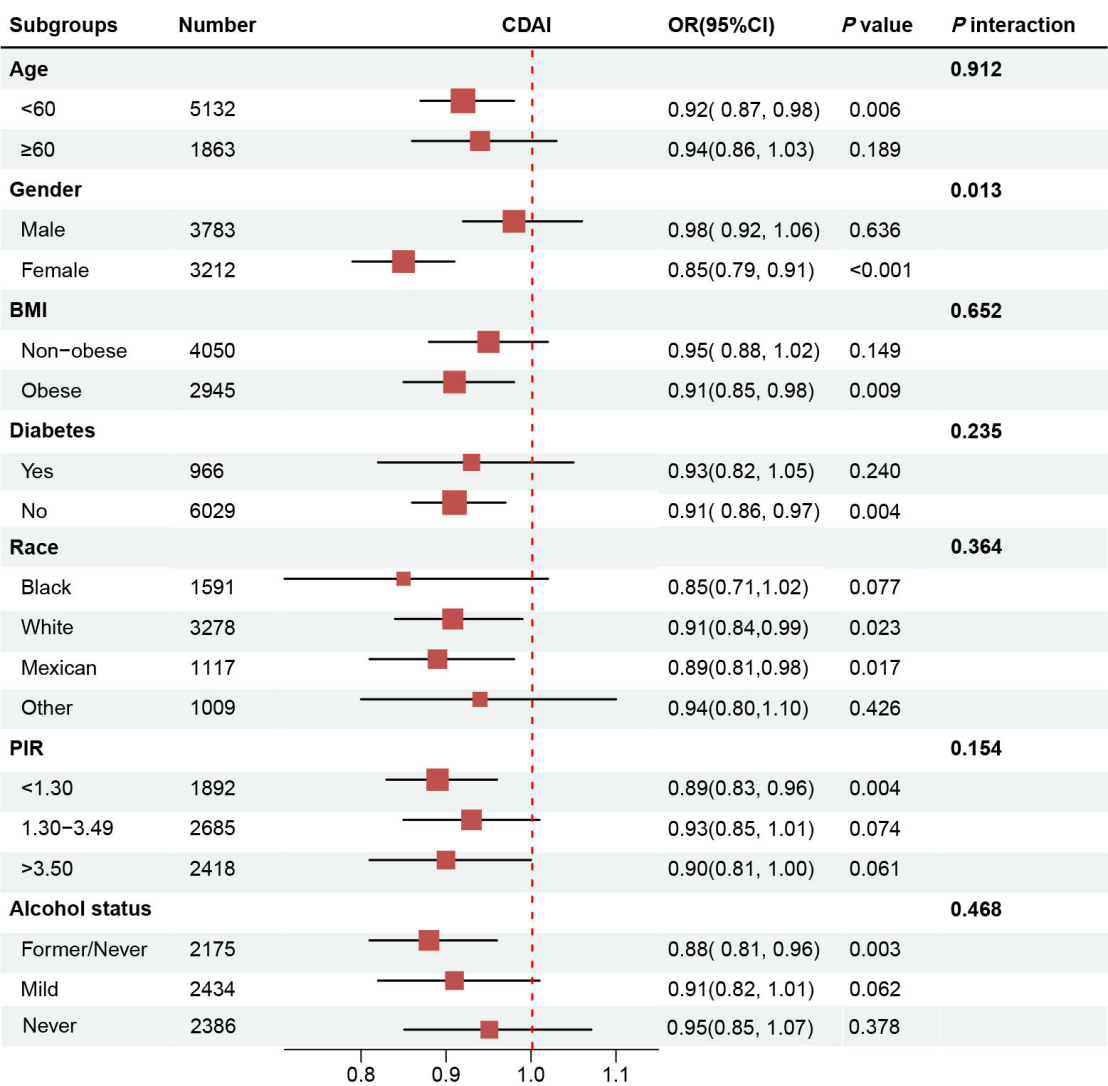
**Subgroup analysis of the association between CDAI and sarcopenia**. Abbreviations: CDAI, composite dietary antioxidant index; PIR, poverty income ratio; OR, odds ratio; CI, confidence interval; BMI, body mass index.

Subgroup analyses stratified by age, gender, BMI, diabetes, race, PIR, and alcohol consumption were performed (Fig. 2). The results indicated that a relatively stronger association between CDAI and sarcopenia among hypertensive adults was found among females, and a significant interaction was observed (*p* interaction <0.050). In the sensitivity analyses, we initially selected 140/90 mmHg as the hypertensive cut-off value to explore the correlation between CDAI and sarcopenia among hypertensive adults. As expected, we found the results were robust by using logistic and linear regression (**Supplementary Table 3**). Next, our result consistently demonstrated that CDAI was related to a decreased risk of sarcopenia among hypertensive adults by using three different hypertension diagnosis criteria (**Supplementary Table 4**).

## 4. Discussion

The present research demonstrated an inverse correlation between CDAI and sarcopenia in hypertensive patients, suggeting a protective effect of CDAI against the progression of sarcopenia, particularly in females. The RCS analysis further indicated a linear trend between CDAI and sarcopenia, suggesting the risk of sarcopenia decreased as CDAI increased. These results underscore the benefits of a diet rich in antioxidants for hypertensive patients in preventing the onset of sarcopenia.

Our results showed that high intake of the antioxidant-rich diet confered a reduced risk of sarcopenia in hypertensive patients, supported by several prior studies. Two large population-based studies have established that adequate vitamin C intake provides protection against sarcopenia [31, 32]. Meanwhile, an observational study by Mulligan *et al*. [33] suggested that consuming more vitamin E is beneficial for musculoskeletal health. Another survey of 1345 participants also discovered an inverse correlation between vitamin E intake and sarcopenia, further demonstrating the advantages of dietary antioxidants for sarcopenia [34]. Additionally, a prospective cohort study data from two community-based cohorts demonstrated that the intake of antioxidant nutrients, particularly carotenoids, is positively related to grip strength and gait speed in elderly individuals, highlighting the impact of an antioxidant diet on muscle mass and physical performance [35]. A previous study also showed that nutritional intervention with antioxidants could enhance muscle mass in antioxidant-deficient mice, indicating that the adverse effects of antioxidant insufficiency can be mitigated by supplementation [36]. Therefore, individuals with high levels of OS, especially hypertensive patients, should modify their dietary patterns to increase CDAI levels to prevent sarcopenia.

Our findings revealed that the risk of skeletal sarcopenia decreased with increasing CDAI in the hypertensive population. Multicenter clinical research involving 2613 participants concluded that hypertension, a chronic age-related metabolic disorder, can lead to diminished muscle function and an elevated risk of sarcopenia [37]. Hypertension is often accompanied by prolonged OS, which can result in muscle injury and disordered muscle protein metabolism [36, 38]. With age, the body’s endogenous antioxidant defence system declines and the excessive accumulation of ROS contributes to oxidative muscle damage. Additionally, mitochondrial dysfunction becomes prominent during muscle aging, a phenomenon linked to dysregulated ROS production and subsequent oxidative damage [39]. Many dietary antioxidants mitigate OS through their bioactive molecules, by integrating metabolic processes, and by regulating gene expression as cellular signaling modulators [40]. Prior literature performed by van Dijk *et al*. [36] demonstrated that supplementation with dietary antioxidants improved mitochondrial dynamics, thereby protecting and enhancing muscle strength and mass in aged mice with antioxidant deficiencies. The maintenance of muscle mass depends on the balance between protein synthesis and degradation [41]. This balance can be disrupted by OS, which accelerates protein degradation and inhibits protein synthesis, thereby promoting skeletal muscle atrophy [36]. OS-related impairment of muscle mass in older adults can be mitigated by antioxidant-rich foods [42]. Several vitamins and carotenoids can improve muscle mass by increasing protein and collagen synthesis while protecting muscles from OS and inflammation [10]. Several studies have already revealed that higher intake of antioxidants, such as carotenoids, vitamin C and E are related to skeletal muscle health [10, 11]. The benefits of healthy dietary patterns in reducing the sarcopenia risk were confirmed in a population-based study of older adults [43]. Micronutrient-rich fruits and vegetables can prevent metabolic acidosis, reduce protein hydrolysis, and decrease amino acid catabolism, thereby contributing to muscle quality and function [44]. In addition, dietary zinc and selenium are also important micronutrients that play a crucial role in combating oxidation; however, high concentrations of these elements can induce toxic and oxidative effects and inhibit antioxidant enzymes [45, 46]. Notably, numerous reports have highlighted that chronic inflammation and IR are also crucial components in the mechanism of sarcopenia. A prior observational study by Luu *et al*. [47] exhibited that CDAI was inversely related to levels of multiple inflammatory biomarkers. Another published research article indicated a reverse correlation between dietary TAC and IR, suggesting the potential effect of an antioxidant diet on improving insulin sensitivity [48]. Therefore, we speculated that adherence to an antioxidant-rich diet may confer potential benefits in improving muscle mass by eliminating the deleterious impacts of chronic inflammation and IR in hypertensive individuals, thereby reducing the risk of sarcopenia.

However, prior research has predominantly focused on the isolated effects of individual antioxidants on muscle mass, neglecting to fully explore the potential interactions and synergistic effects among different antioxidants. The CDAI provides a comprehensive evaluation of an individual’s overall antioxidant intake by combining various dietary components, including vitamins, and other antioxidants. This integrated approach reflects the combined effects of multiple antioxidants in the diet, rather than focusing on a single nutrient. CDAI mitigates potential biases and errors inherent in single nutrient studies by integrating the effects of multiple antioxidants, thereby enhancing the reliability of the results. CDAI offers a more accurate reflection of antioxidant intake in daily diets, while individual antioxidants are typically confined to specific foods or supplements.

According to our subgroup analysis, it was noted that an inverse association of CDAI and sarcopenia was found only in females but not males. This phenomenon may be owing to the distinctions in endogenous sex hormones. Estrogen, a hormone predominantly found in females, is known to have an antioxidant-like impact, by reducing ROS production and stimulating the upregulation of genes encoding antioxidant enzymes [49, 50]. Moreover, estrogens contain phenol hydroxyl and methyl groups, which confer antioxidant properties by scavenging oxygen free radicals [51]. Conversely, androgens, which are predominantly found in males, are known to induce OS by facilitating the generation of ROS, significantly diminishing the benefits of an antioxidant-rich diet on muscle health in men [52]. More importantly, females generally exhibit superior dietary habits compared to males, consuming more micronutrient-rich foods such as fruits and vegetables, and employing healthier cooking and processing methods [53]. Thus, females with hypertension may derive more benefit from a high antioxidant diet than males with hypertension.

Our research has several strengths. Firstly, our data was established from the NHANES database, a large-scale national investigation, which helped ensure the reliability of our results. Secondly, by counting dietary CDAI, our research firstly clarified the negative correlation between sarcopenia and overall dietary antioxidant capacity in hypertensive individuals, revealing that higher CDAI serves as a protective factor against sarcopenia. Thirdly, the stability of our results was confirmed by sensitivity analyses performed by different approaches.

However, several limitations of our research should also be considered. Firstly, given the cross-sectional nature of the data, the causal effect cannot be established between CDAI and sarcopenia. Secondly, since the NHANES data did not distinguish between primary and secondary sarcopenia, and the diagnosis was based solely on muscle mass without incorporating grip strength, which may limit the generalizability of our findings to all types of sarcopenia. Thirdly, dietary data was collected through 24 hour recall interviews, which might lead to recall bias and may not accurately reflect the actual dietary intake. Additionally, the 24 hour dietary recall method may not capture habitual dietary patterns, introducing potential measurement biases in the dietary assessment. Fourthly, the CDAI was calculated based solely on baseline dietary data, which may not accurately reflect long-term dietary patterns. Moreover, the singular diagnostic criterion for sarcopenia based solely on muscle mass without considering muscle strength or physical performance may limit the generalizability of the results. Finally, despite adjusting for known conventional variables, there may still be unmeasured confounders that could affect our results, such as physical activity levels and chronic disease burden, which were not accounted for in our analysis. Future prospective cohort studies are required to shed more light on our results.

## 5. Conclusions

The present study, based on data from NHANES, detected an inverse connection between CDAI and sarcopenia in hypertensive adults, suggesting that an antioxidant-rich diet may offer a beneficial impact and serve as an effective method of preventing sarcopenia for hypertensive patients.

## Availability of Data and Materials

Data described in the manuscript are publicly and freely available without restriction at https://www.cdc.gov/nchs/nhanes/index.htm.

## Supplementary Material

Supplementary material associated with this article can be found, in the online version, at https://doi.org/10.31083/RCM27138.
