# Supplementary material for: Association of Dietary Antioxidant Potential with Sarcopenia in Hypertension
Source: Rev Cardiovasc Med. 2025 Apr 24;26(4):27138. doi: 10.31083/RCM27138 (PMC12059757; doi:10.31083/RCM27138)
Supplement: Supplementary file 1 [file 2153-8174-26-4-27138-s1.zip › RCM27138-Supplementary Material-V2.docx]

**Supplementary Table 1.** The formula for CDAI calculation

| Index | Formula |
| --- | --- |
| CDAI | $CDAI=\sum_{i=1}^{n=6} \frac{Individual Intake-Mean}{\mathrm{SD}}$ |

Abbreviation: CDAI, composite dietary antioxidant index; SD, standard error.

**Supplementary Table 2.** Detailed description and options of covariables in the National Health and Nutrition Examination Survey (NHANES) database.

| Covariables | Description in NHANES |
| --- | --- |
| Age | Age |
| Gender | Gender (Female,male) |
| Race | Race (Non-Hispanic Black, Non-Hispanic White, Mexican American, and other races) |
| Education level | Education level（less than 9th grade, 9th-11th grade, high school, some college, and college or above） |
| Poverty income ratio | A ratio of family income to poverty（<1.30, 1.30-3.49, and ≥ 3.50） |
| Marital status | Marital status ( married, unmarried, and others (widowed, divorced, and separated)) |
| Energy | The total daily energy intake in the NHANES database represented the total number of kilocalories consumed from all foods and beverages by a participant over a 24-hour period. |
| White blood cell | WBC was obtained from NHANES laboratory data. |
| Uric acid | Uric acid was obtained from NHANES laboratory data. |
| Blood urea nitrogen | Blood urea nitrogen was obtained from NHANES laboratory data. |
| Smoke status | Participants were asked whether they smoked at least 100 cigarettes in life (yes and no). |
| Cardiovascular disease | Cardiovascular Disease included coronary heart disease, congestive heart failure, heart attack, stroke, angina. Patients were asked whether they have been told they had coronary heart disease, congestive heart failure, heart attack, stroke, angina. (yes and no). |
| Chronic kidney disease | We utilized the Chronic Kidney Disease Epidemiology Collaboration equation to compute the estimated glomerular filtration rate and assess Chronic kidney disease. |
| Diabetes | Diabetes was defined as the diagnosis told by doctors or current use of diabetes medications or insulin. |
| Hyperlipidemia | Participants were defined as having hyperlipidemia if they had a triglyceride level ≥150 mg/dL,or total cholesterol level >200 mg/dL,or low-density lipoprotein cholesterol ≥130mg/dL，or high-density lipoproteins <40mg/dL, or took lipid‐lowering medication. |
| Alcohol status | Alcohol status was categorized as never (< 12 drinks in a lifetime), former (≥12 drinks in 1 year and did not drink last year), mild drinker (≤ 1 drink/day for women or ≤ 2 drinks/day for men), and heavy drinker (≥ 2 drinks/day for women or ≥3 drinks/day for men). |

Abbreviations: NHANES, National Health and Nutrition Examination Survey

**Supplementary Table 3.** Association between CDAI and sarcopenia risk among hypertensive adults using a hypertensive cut-off value of 140/90 mmHg (n=5015).

| Variables | Model 1 | | Model 2 | | Model 3 | |
| --- | --- | --- | --- | --- | --- | --- |
|  | β/OR (95%CI) | *P* value | β/OR (95%CI) | *P* value | β/OR (95%CI) | *P* value |
| **ALM (kg)** |  |  |  |  |  |  |
| Continuous | 0.15(0.07, 0.23) | <0.001 | 0.15(0.10, 0.20) | <0.001 | 0.07(0.03, 0.11) | <0.001 |
| Categories |  |  |  |  |  |  |
| Q1 | Reference |  | Reference |  | Reference |  |
| Q2 | 1.09(0.34, 1.84) | 0.005 | 0.63(0.15, 1.10) | 0.010 | 0.28(-0.12, 0.69) | 0.170 |
| Q3 | 1.64(0.84, 2.44) | <0.001 | 1.04(0.55, 1.53) | <0.001 | 0.50(0.07, 0.93) | 0.022 |
| Q4 | 1.76(0.97, 2.54) | <0.001 | 1.62(1.13, 2.11) | <0.001 | 0.81(0.29, 1.33) | 0.003 |
| *P* for trend | <0.001 |  | <0.001 |  | 0.002 |  |
| Per 1-SD increase | 0.55(0.27, 0.83) | <0.001 | 0.56(0.39, 0.74) | <0.001 | 0.27(0.12, 0.41) | <0.001 |
| **Sarcopenia** |  |  |  |  |  |  |
| Continuous | 0.88(0.85,0.92) | <0.001 | 0.88(0.84, 0.92) | <0.001 | 0.89(0.83, 0.96) | 0.002 |
| Categories |  |  |  |  |  |  |
| Q1 | Reference |  | Reference |  | Reference |  |
| Q2 | 0.77(0.59,1.01) | 0.057 | 0.71(0.54, 0.94) | 0.015 | 0.71(0.51, 0.98) | 0.036 |
| Q3 | 0.54(0.38,0.75) | <0.001 | 0.49(0.34, 0.70) | <0.001 | 0.46(0.30, 0.71) | <0.001 |
| Q4 | 0.30(0.21,0.43) | <0.001 | 0.29(0.20, 0.41) | <0.001 | 0.28(0.17, 0.46) | <0.001 |
| *P* for trend | <0.001 |  | <0.001 |  | <0.001 |  |
| Per 1-SD increase | 0.63(0.54,0.74) | <0.001 | 0.62(0.53, 0.72) | <0.001 | 0.66(0.50, 0.86) | 0.002 |

Model 1: Adjusted for age

Model 2: Adjusted for age, gender, and race.

Model 3: Adjusted for age, gender, race, marital status, education level, poverty income ratio, BMI, smoke, alcohol status, CVD, CKD, diabetes, uric acid, BUN, WBC, hyperlipidemia, and energy.

Abbreviations: Q, quartiles; SE, standard error; CDAI, composite dietary antioxidant index; PIR, poverty income ratio; WBC, white blood cell; BUN, blood urea nitrogen; CVD, cardiovascular disease; CKD, chronic kidney disease; BMI, body mass index; ALM, appendicular lean mass; ORs, odds ratios; CIs, confidence intervals; SD, standard deviation.

**Supplementary Table 4.** Association between CDAI and sarcopenia risk among hypertensive adults by using three diagnosis criteria.

|  | Model 1 | | Model 2 | | Model 3 | |
| --- | --- | --- | --- | --- | --- | --- |
|  | **Hypertension diagnosed by blood pressure values (n = 5364)** | | | | | |
| **ALM (kg)** | β(95%CI) | *P* value | β(95%CI) | *P* value | β(95%CI) | *P* value |
| Continuous | 0.08(0.02, 0.14) | 0.011 | 0.11(0.07, 0.15) | <0.001 | 0.06(0.03, 0.10) | <0.001 |
| Categories |  |  |  |  |  |  |
| Q1 | Reference |  | Reference |  | Reference |  |
| Q2 | 0.7(0.08, 1.32) | 0.027 | 0.58(0.18, 0.97) | 0.005 | 0.19(-0.12, 0.50) | 0.226 |
| Q3 | 1.13(0.27, 1.99) | 0.011 | 0.84(0.28, 1.41) | 0.004 | 0.30(-0.10, 0.69) | 0.137 |
| Q4 | 1.28(0.63, 1.93) | <0.001 | 1.26(0.87, 1.65) | <0.001 | 0.60(0.23, 0.97) | 0.002 |
| *P* for trend |  |  |  |  |  |  |
| Per 1-SD increase | 0.29(0.07, 0.51) | 0.011 | 0.4(0.25, 0.55) | <0.001 | 0.23(0.10, 0.36) | <0.001 |
| **Sarcopenia** | OR (95%CI) | *P* value | OR (95%CI) | *P* value | OR (95%CI) | *P* value |
| Continuous | 0.89(0.85,0.92) | <0.001 | 0.88(0.84, 0.92) | <0.001 | 0.92(0.86, 0.98) | 0.007 |
| Categories |  |  |  |  |  |  |
| Q1 | Reference |  | Reference |  | Reference |  |
| Q2 | 0.70(0.54,0.90) | 0.007 | 0.67(0.52, 0.87) | 0.003 | 0.73(0.54, 1.01) | 0.054 |
| Q3 | 0.58(0.41,0.81) | 0.001 | 0.55(0.40, 0.77) | <0.001 | 0.62(0.39, 0.98) | 0.042 |
| Q4 | 0.31(0.22,0.43) | <0.001 | 0.30(0.21, 0.42) | <0.001 | 0.36(0.21, 0.64) | <0.001 |
| *P* for trend | <0.001 |  | <0.001 |  | 0.001 |  |
| Per 1-SD increase | 0.64(0.54,0.75) | <0.001 | 0.63(0.53, 0.74) | <0.001 | 0.73(0.57, 0.92) | 0.007 |
|  | **Hypertension diagnosed by physician (n =4013)** | | | | | |
| **ALM (kg)** | β(95%CI) | *P* value | β(95%CI) | *P* value | β(95%CI) | *P* value |
| Continuous | 0.18(0.10, 0.26) | <0.001 | 0.17(0.11, 0.22) | <0.001 | 0.08(0.04, 0.12) | <0.001 |
| Categories |  |  |  |  |  |  |
| Q1 | Reference |  | Reference |  | Reference |  |
| Q2 | 0.87(0.00, 1.74) | 0.050 | 0.55(0.01, 1.10) | 0.048 | 0.10(-0.30, 0.51) | 0.610 |
| Q3 | 1.71(0.85, 2.58) | <0.001 | 1.32(0.75, 1.90) | <0.001 | 0.47(0.10, 0.84) | 0.014 |
| Q4 | 2.00(1.17, 2.84) | <0.001 | 1.78(1.24, 2.32) | <0.001 | 0.72(0.32, 1.12) | <0.001 |
| *P* for trend | <0.001 |  | <0.001 |  | <0.001 |  |
| Per 1-SD increase | 0.67(0.37, 0.97) | <0.001 | 0.62(0.42, 0.82) | <0.001 | 0.31(0.16, 0.46) | <0.001 |
| **Sarcopenia** | OR (95%CI) | *P* value | OR (95%CI) | *P* value | OR (95%CI) | *P* value |
| Continuous | 0.89(0.85,0.93) | <0.001 | 0.88(0.84, 0.92) | <0.001 | 0.89(0.81, 0.96) | 0.006 |
| Categories |  |  |  |  |  |  |
| Q1 | Reference |  | Reference |  | Reference |  |
| Q2 | 0.74(0.54,1.01) | 0.060 | 0.69(0.50, 0.95) | 0.024 | 0.63(0.43, 0.93) | 0.021 |
| Q3 | 0.55(0.38,0.79) | 0.002 | 0.50(0.34, 0.72) | <0.001 | 0.40(0.25, 0.63) | <0.001 |
| Q4 | 0.31(0.21,0.45) | <0.001 | 0.29(0.20, 0.43) | <0.001 | 0.24(0.14, 0.42) | <0.001 |
| *P* for trend | <0.001 |  | <0.001 |  | <0.001 |  |
| Per 1-SD increase | 0.64(0.55,0.76) | <0.001 | 0.62(0.53, 0.74) | <0.001 | 0.63(0.46, 0.87) | 0.006 |
|  | **Hypertension diagnosed by anti-hypertensive drugs (n =3167)** | | | | | |
| **ALM (kg)** | β(95%CI) | *P* value | β(95%CI) | *P* value | β(95%CI) | *P* value |
| Continuous | 0.18(0.09, 0.27) | <0.001 | 0.18(0.12, 0.24) | <0.001 | 0.06(0.02, 0.11) | 0.006 |
| Categories |  |  |  |  |  |  |
| Q1 | Reference |  | Reference |  | Reference |  |
| Q2 | 0.72(-0.27, 1.72) | 0.153 | 0.56(-0.06, 1.18) | 0.077 | 0.17(-0.26, 0.61) | 0.430 |
| Q3 | 1.79(0.87, 2.71) | <0.001 | 1.29(0.64, 1.94) | <0.001 | 0.37(-0.01, 0.76) | 0.059 |
| Q4 | 1.95(1.05, 2.86) | <0.001 | 1.91(1.27, 2.55) | <0.001 | 0.70(0.22, 1.19) | 0.005 |
| *P* for trend | <0.001 |  | <0.001 |  | 0.003 |  |
| Per 1-SD increase | 0.68(0.35, 1.02) | <0.001 | 0.68(0.46, 0.90) | <0.001 | 0.24(0.07, 0.40) | 0.006 |
| **Sarcopenia** | OR (95%CI) | *P* value | OR (95%CI) | *P* value | OR (95%CI) | *P* value |
| Continuous | 0.91(0.87,0.95) | <0.001 | 0.90(0.86, 0.95) | <0.001 | 0.91(0.84, 0.99) | 0.025 |
| Categories |  |  |  |  |  |  |
| Q1 | Reference |  | Reference |  | Reference |  |
| Q2 | 0.78(0.54,1.11) | 0.163 | 0.72(0.49, 1.04) | 0.077 | 0.68(0.45, 1.04) | 0.078 |
| Q3 | 0.58(0.38,0.88) | 0.012 | 0.52(0.33, 0.81) | 0.004 | 0.45(0.27, 0.73) | 0.001 |
| Q4 | 0.39(0.26,0.58) | <0.001 | 0.36(0.23, 0.55) | <0.001 | 0.32(0.19, 0.54) | <0.001 |
| *P* for trend | <0.001 |  | <0.001 |  | <0.001 |  |
| Per 1-SD increase | 0.69(0.58,0.83) | <0.001 | 0.67(0.55, 0.81) | <0.001 | 0.70(0.51, 0.96) | 0.025 |

Model 1: Adjusted for age

Model 2: Adjusted for age, gender, and race.

Model 3: Adjusted for age, gender, race, marital status, education level, poverty income ratio, BMI, smoke, alcohol status, CVD, CKD, diabetes, uric acid, BUN, WBC, hyperlipidemia, and energy.

Abbreviations: Q, quartiles; SE, standard error; CDAI, composite dietary antioxidant index; PIR, poverty income ratio; WBC, white blood cell; BUN, blood urea nitrogen; CVD, cardiovascular disease; CKD, chronic kidney disease; BMI, body mass index; ALM, appendicular lean mass; ORs, odds ratios; CIs, confidence intervals; SD, standard deviation.

**Supplementary Fig 1.** Flow chart of the study design.


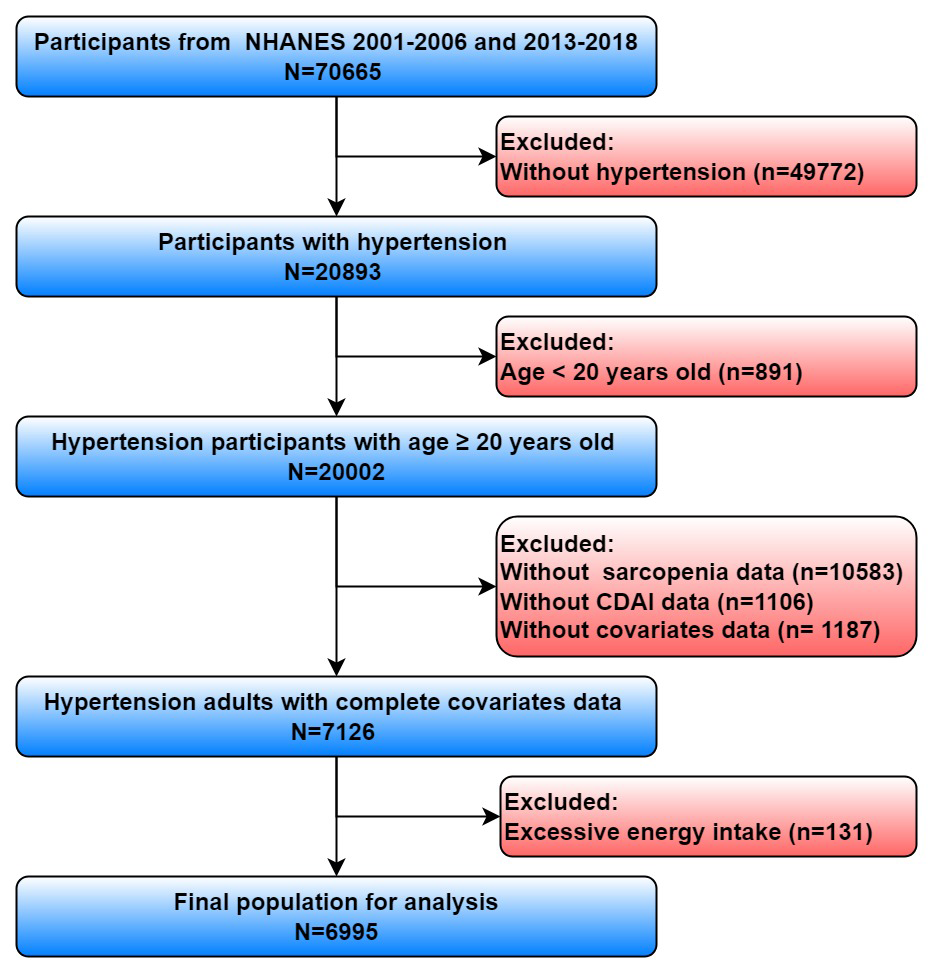


Abbreviations: NHANES, National Health and Nutrition Examination Survey; CDAI, composite dietary antioxidant index

**Supplementary Fig 2.** Restricted cubic spline analysis with multivariate-adjusted association between individual antioxidants and sarcopenia.


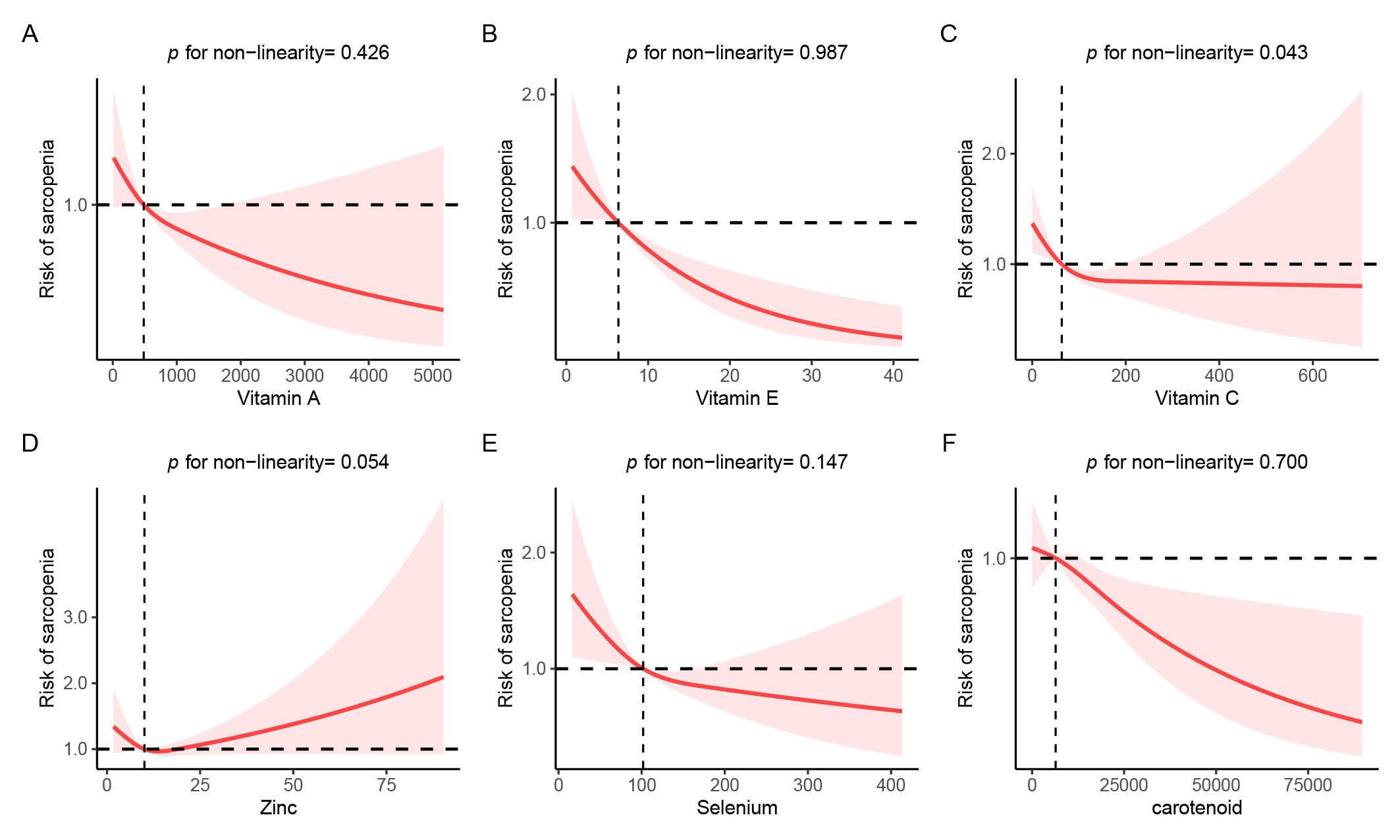


1. RCS analysis between vitamin A and sarcopenia risk. (B) RCS analysis between vitamin E and sarcopenia risk. (C) RCS analysis between vitamin C and sarcopenia risk. (D) RCS analysis between zinc and sarcopenia risk. (E) RCS analysis between selenium and sarcopenia risk. (F) RCS analysis between carotenoid and sarcopenia risk.

Abbreviations: RCS, restricted cubic spline; CDAI, composite dietary antioxidant index.
