# Supplementary figures and images for: Association of Dietary Antioxidant Potential with Sarcopenia in Hypertension
Source: Rev Cardiovasc Med. 2025 Apr 24;26(4):27138. doi: 10.31083/RCM27138 (PMC12059757; doi:10.31083/RCM27138)

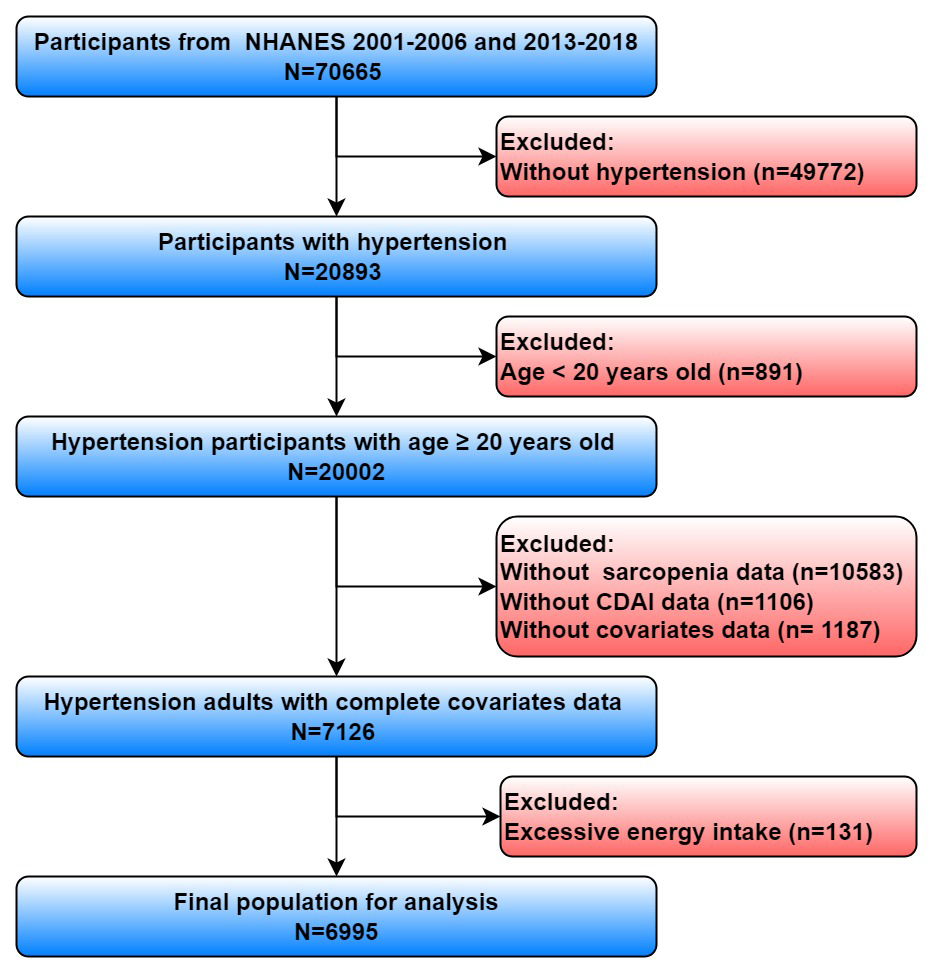

Supplement: Supplementary file 1 [file 2153-8174-26-4-27138-s1.zip › Supplementary Fig 1.tif]

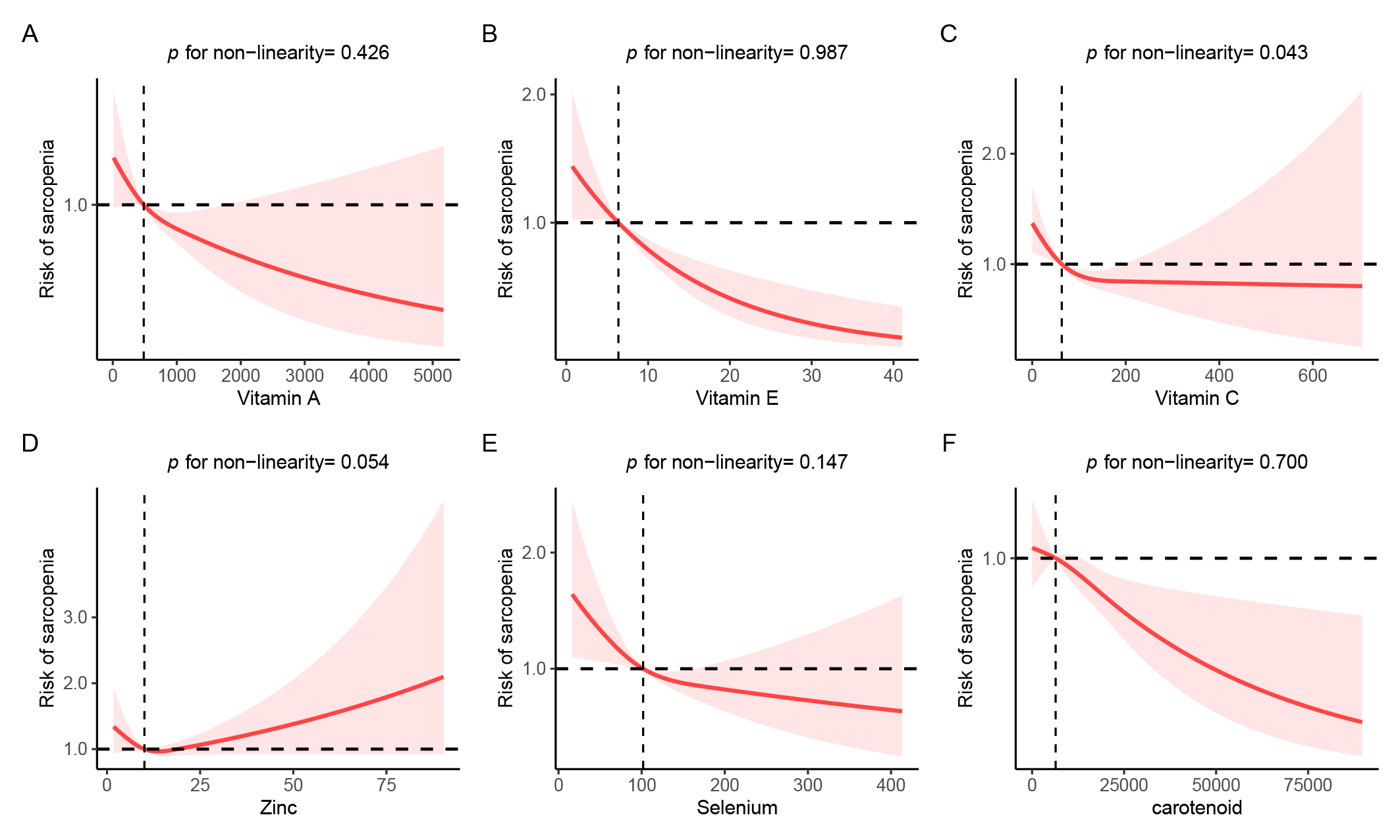

Supplement: Supplementary file 1 [file 2153-8174-26-4-27138-s1.zip › Supplementary Fig 2.tif]
